# Direct observation of lithium metal dendrites with ceramic solid electrolyte

**DOI:** 10.1038/s41598-020-75456-0

**Published:** 2020-10-27

**Authors:** Maryam Golozar, Andrea Paolella, Hendrix Demers, Sylvio Savoie, Gabriel Girard, Nicolas Delaporte, Raynald Gauvin, Abdelbast Guerfi, Henning Lorrmann, Karim Zaghib

**Affiliations:** 1 Center of Excellence in Transportation Electrification and Energy Storage, Hydro-Québec, Varennes, QC J0L 1N0 Canada; 2 Department of Mining and Materials Engineering, McGill University, Montreal, QC H3A 0C5 Canada; 3 Fraunhofer-Institut für Silicatforschung ISC, Neunerplatz 2, 97082 Würzburg, Germany

**Keywords:** Materials for energy and catalysis, Energy storage, Energy, Chemistry, Energy science and technology, Materials science, Nanoscience and technology

## Abstract

Dendrite formation, which could cause a battery short circuit, occurs in batteries that contain lithium metal anodes. In order to suppress dendrite growth, the use of electrolytes with a high shear modulus is suggested as an ionic conductive separator in batteries. One promising candidate for this application is Li_7_La_3_Zr_2_O_12_ (LLZO) because it has excellent mechanical properties and chemical stability. In this work, in situ scanning electron microscopy (SEM) technique was employed to monitor the interface behavior between lithium metal and LLZO electrolyte during cycling with pressure. Using the obtained SEM images, videos were created that show the inhomogeneous dissolution and deposition of lithium, which induce dendrite growth. The energy dispersive spectroscopy analyses of dendrites indicate the presence of Li, C, and O elements. Moreover, the cross-section mapping comparison of the LLZO shows the inhomogeneous distribution of La, Zr, and C after cycling that was caused by lithium loss near the Li electrode and possible side reactions. This work demonstrates the morphological and chemical evolution that occurs during cycling in a symmetrical Li–Li cell that contains LLZO. Although the superior mechanical properties of LLZO make it an excellent electrolyte candidate for batteries, the further improvement of the electrochemical stabilization of the garnet–lithium metal interface is suggested.

## Introduction

Metallic lithium is a potential anode material for high energy density Li-ion batteries because of its high capacity (3860 mAh g^−1^ for the reduced form)^1^. Lithium anodes, however, undergo dendrite formation during cycling that can increase the risk of battery short circuit^2^. To overcome this limitation, one technique is the use of solid electrolytes with a high shear modulus to withstand perforations by dendrites and thus prevent battery short circuit. Monroe and Newman^3^ have reported that a shear modulus that is twice that of Li can suppress dendrite growth. Recently, the use of ceramics as solid electrolytes in all-solid Li metal batteries have attracted interest because of their high shear modulus. The failure and short circuit of batteries that contain ceramic electrolytes, however, have been reported^4–7^. One interesting electrolyte material for this application is Li_7_La_3_Zr_2_O_12_ (LLZO) because it has (a) a high voltage stability (up to 5 V), (b) high conductivity at room temperature (> 1 × 10^−4^ S/cm), (c) low kinetic reactivity with Li, and (d) high shear modulus (approximately 55 GPa)^8–12^. Although LLZO satisfies the Monroe and Newman criteria and has exhibited promising properties, the failure of batteries that contain this electrolyte has been reported. Aguesse et al.^5^ indicated the electrochemical collapse of batteries that contain LLZO electrolytes at room temperature as a result of Li metal formation in the LLZO during cycling. It is also found that lithium metal propagation through this electrolyte occurs through the grain boundaries^6^. Shen et al.^13^ correlated the battery short circuit to the interconnected pores in the LLZO. Basappa et al.^14^ solved the pore interconnectivity problem by modifying the grain boundaries.

In order to fully understand the behavior of garnet LLZO during cycling, further investigations are necessary. This work focuses on observing the failure behavior of the cell containing LLZO electrolyte including conducting chemical analysis on the dendrites. In this in situ study, scanning electron microscopy (SEM) is employed to monitor the behavior of Li surface and Li_7_La_3_Zr_2_O_12_ during cycling. Videos are constructed using a multitude of subsequent SEM images that exhibit the morphological evolution during cycling. Energy dispersive spectroscopy (EDS) is also employed to conduct a chemical analysis. The *set up* and the experiments of this work are designed in a way to push the cell to its limits and to observe dissolution and deposition behavior and to observe dendrites. The results show inhomogeneous dissolution and deposition of lithium, leading to the formation of mossy and needle morphology dendrites. The chemical analysis of dendrites shows that they are mainly composed of Li_2_CO_3_ and Li_x_C_y_, and Li_2_O. The chemical comparison of the LLZO cross-sections before and after cycling also shows the inhomogeneous distribution of Zr, La, and C after cycling as a result of lithium loss reaction near the lithium metal electrode.

## Materials and methods

### Ceramic and symmetrical Li–LLZO–Li cell preparation

Symmetrical Li cells with Li_7_La_3_Zr_2_O_12_ (LLZO) solid electrolytes were used in this study. Following our previous reports^15,16^, the gallium-doped LLZO electrolyte is synthesized by mixing 5.92 g of Li_2_CO_3_, 11.39 g of La_2_O_3_, 5.77 g of ZrO_2_ and 0.56 g of Ga_2_O_3_ in planetary mill with ZrO_2_ balls under air atmosphere and then the mixture is annealed in tubular furnace on graphite (or zirconia or alumina) boat. The temperature was increased from room temperature to 700 °C and then the synthesis temperature was increased up to 950 °C and kept for 2 h keeping N_2_ gas flowing. Finally, the powder was cooled down. The final powder is cold pressed at 100 MPa and annealed in air atmosphere at 1100 °C for 10 h.The final ceramic pellet has a thickness of 1 mm and an ionic conductivity at room temperature and 80 °C of 6 × 10^−4^ S/cm and 2 × 10^−3^ S/cm respectively (see Figure S1). The LLZO pellet shows a density of 99%. A plane view cell assembly and set up were used in this work^17^. Lithium metal electrodes (Hydro-Quebec) that are 34 µm thick are pressed on both sides of the electrolyte. The surface of the Li electrode is covered with residuals of polyether oxide^18^ used as lubricant for the thin film fabrication. To induce dendrite growth, the Li electrode facing the SEM electron beam, which is monitored during cycling, has a smaller area than the LLZO with area of 1.33 cm^2^ that creates edge effect^17^. A copper spring was used as contact electrode and to apply pressure on the Li film to push it on the LLZO electrolyte and to make good contact between them^17,19^. The lower Li electrode has the same area as LLZO and rest on the flat aluminum sample holder, which acts as a contact electrode and push the lower Li electrode on the LLZO electrolyte. The electrodes were connected to the cycler using an electric feedthrough installed in one of the SEM port. A schematic of the set up is shown in Figure S2. The cell is assembled in the glove box and thereafter transferred to the SEM using an airtight sample holder.

### In situ cycling and post-mortem analysis

A TESCAN scanning electron microscope (Mira 3) was employed to perform the in situ imaging of cells during cycling. In situ set up, transfer holder, and acquisition software were designed and developed at Hydro-Québec. Beam energy of 5.00 kV was used for imaging and analysis. Also during cycling, images were obtained every 30 min from different areas of the upper Li and LLZO and the beam was blanked during the time that no images were obtained to minimize the exposure of each region to the beam. Thereafter these images were used for constructing videos to demonstrate the behavior of the cell during Li shuttling. Ex situ analyses were conducted using a focused ion beam scanning electron microscope (FIB-SEM) (TESCAN Lyra 3 GT FIB-SEM) with a gallium ion source-focused ion beam. Chemical analyses were conducted using a windowless EDS detector with extreme electronics (Oxford Instrument) that enables the detection of Li^20^.

## Results and discussion

**Figure 1:**
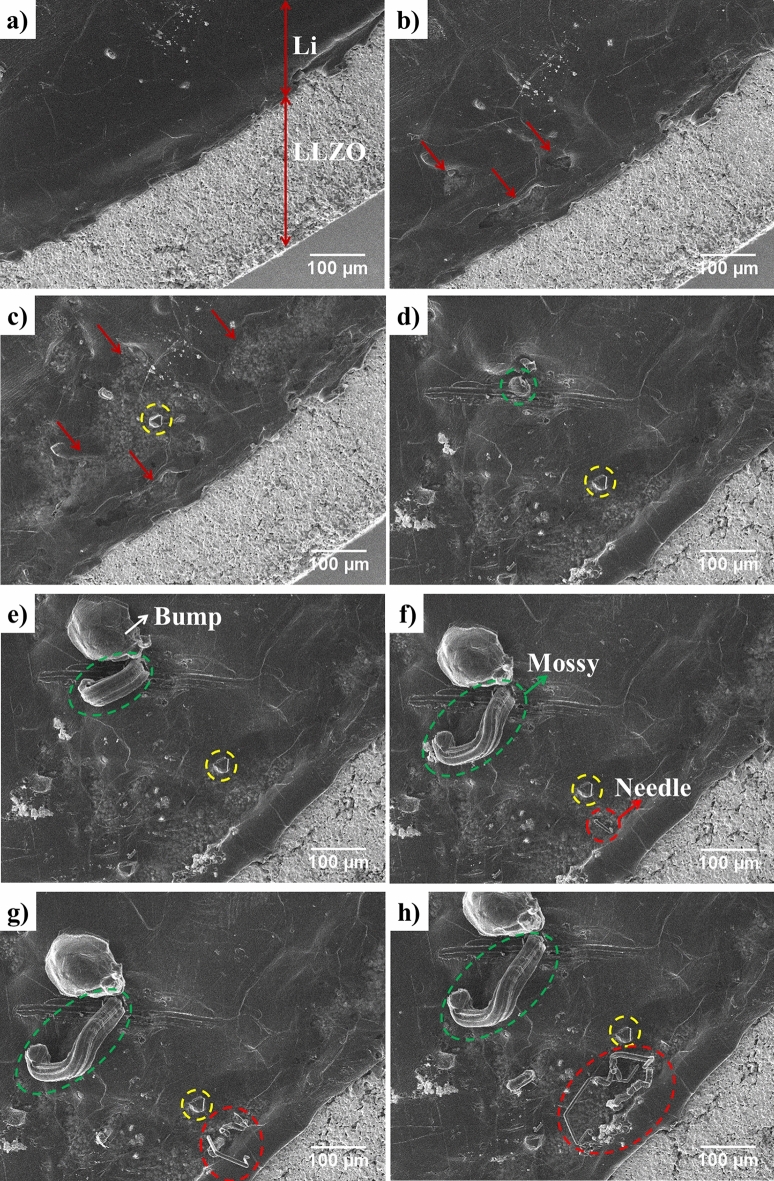
Obtained cell SEM images corresponding to Video S1; (**a**) Li and LLZO (at − 0.0036 V and − 7.5 µA cm^−2^) at the beginning of cycling; (**b**) beginning of Li thinning (at − 0.0057 V and − 7.5 µA cm^−2^) after 50 h cycling ; (**c**) further Li thinning and formation of a bump on Li indicated by yellow dotted circle (at − 0.0018 V and + 15 µA cm^−2^) after 94 h cycling; (**d**) initiation of the extrusion of mossy dendrite indicated by green dotted circle (at − 0.0022 V and + 15 µA cm^−2^) after 97 h cycling; (**e**) further extrusion of mossy dendrite and formation of an adjacent bump morphology (at − 0.0022 V and + 15 µA cm^−2^) after 110 h cycling; (**f**) mossy dendrite and initiation of needle dendrite formation indicated by red dotted circle (at − 0.0022 V and + 15 µA cm^−2^) after 119 h cycling; (**g**) further growth of mossy and needle dendrites (at − 0.0034 V and + 15 µA cm^−2^) after 124 h cycling; (**h**) mossy and needle dendrites and bump morphology on Li surface (at − 0.0018 V and + 15 µA cm^−2^) after 128 h cycling. A small shift in images was observed because of specimen drifting during cycling that can be monitored by following the yellow dotted circle.

**Figure 2:**
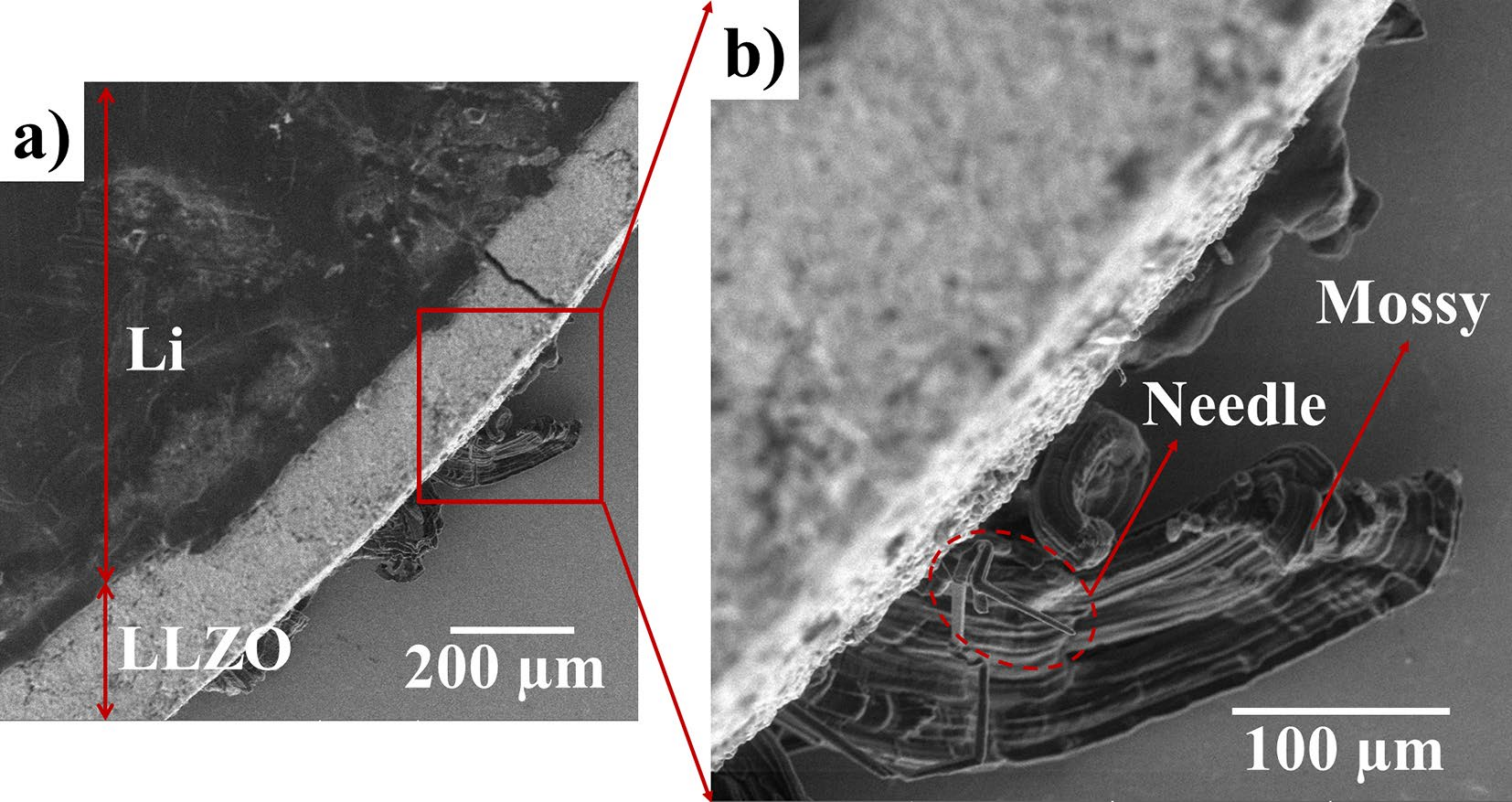
SEM images of the cell with dendrites with mossy and needle morphologies on Li at the bottom side of LLZO formed when applied current has negative values.

Figure 1a shows the surface of Li and LLZO before cycling. The cell is cycled in the microscope at 80 °C to reach the ionic conductivity of 2 × 10^−3^ S/cm. Figure S3 shows the cycling curve of the cell: first, a − 7.5 µA cm^−2^ current density was applied to induce Li metal deposition on the Li electrode at the bottom of LLZO that does not face the electron beam. After 1 day of cycling, the current density was changed to − 15 µA cm^−2^. After 4 days, the current density was increased to $+$ 15 µA cm^−2^; Li deposition occurs on the Li electrode facing the electron beam. The increase in the current density was done to monitor the failure of the cell faster and at extreme conditions. Figure 2 depicts two dendrite morphologies of needle and mossy on the bottom Li electrode that were formed during Li deposition on the bottom side. These dendrites could have been initiated from the defects such as the grain boundaries, regions covered with thin solid electrolyte interphase (SEI) layer, and possible contaminations^19,21^. The fact that these dendrites are observed in this specific region of the bottom Li electrode from the top view could be due to possible low pressure in this area that allows for further dendrite growth outwards the cell. The SEM images in Fig. 1 correspond to those of Video S1, which demonstrates the behavior of the cell from the beginning to end of cycling. The phenomena observed during cycling include Li thinning, formation of mossy morphology dendrite, bump morphology deposition, formation of needle morphology dendrite, and change in the LLZO chemical composition. Figure 1b,c show the thickness evolution of some regions of Li after 50 and 94 h of cycling, respectively (corresponding to − 7.5 µA cm^−2^, at − 0.0057 V and + 15 µA cm^−2^, at − 0.0018 V respectively). Figure 1d shows the initiation of a mossy dendrite morphology after 97 h of cycling (corresponding to + 15 µA cm^−2^, at − 0.0022 V). In addition, Fig. 1e illustrates the further growth of a mossy dendrite and the formation of a bump beside it after 110 h of cycling (corresponding to + 15 µA cm^−2^, at − 0.0022 V). Figure 1f depicts an emerging needle dendrite after 119 h of cycling on the region where Li thinning was observed (corresponding to + 15 µA cm^−2^, at − 0.0022 V). Figure 1g,h show the further growth of dendrites on Li after 124 and 128 h of cycling, respectively (corresponding to + 15 µA cm^−2^, at − 0.0034 V and + 15 µA cm^−2^, at − 0.0018 V respectively). The many spiked that are observed in the cycling curve is due to the formation of dendrites and the loss of Li as the result of thinning of Li electrode that could lower the contact between the Li and the LLZO electrodes. The goal of this work is to study the failure of the cell. Therefore the cell is push to the extreme in order to be able to collect data and images of the cell in a shorter period of time using the SEM. These phenomena are further discussed in detail below.

### Thinning of Li electrode

For reference, a dense Li film in theory (homogenous film dissolution) loses approximately 5 µm per 1 mAh cm^−2^ of charge. The thickness of some regions in the Li electrode facing the electron beam was significantly reduced during cycling (Figs. 1, 2). Figure S4 shows three SEM images at higher magnification, focused on the thinned regions. Figure S4b shows the diameter and thickness of a thinned region after 4 days of cycling. This thickness reduction was correlated to Li metal dissolution, which is more uniform compared to that in Li metal polymer batteries reported by Golozar et al.^18^ and Hovington et al.^1^. In batteries that use polymer electrolytes, a more local dissolution starting from the grain boundaries is observed^19^. In the case of LLZO, however, the dissolution of Li was observed in larger areas; in some cases containing a few neighboring grains at different parts of the Li electrode. This may be because of the higher Li^+^ transfer number in the LLZO than in polymers^22^ and elimination of salt decomposition reported in polymer batteries^19,23^. Although Li dissolution was more uniform in these cells, the change in Li thickness during the dissolution was still inhomogeneous on the electrode surface. To further investigate this phenomenon, the windowless EDS detector was employed to conduct mappings of the Li surface. Lithium has an X-ray energy of 52 eV, which is too low to be detected with a standard EDS detector^20,24^; on the contrary, a windowless detector can detect Li. Different from a standard detector, this detector (a) does not have a window, which overcomes the absorption of low energy X-ray limitations and (b) it has low-noise extreme electronics, which increases its detection capabilities of low energy X-ray^20^.

**Figure 3:**
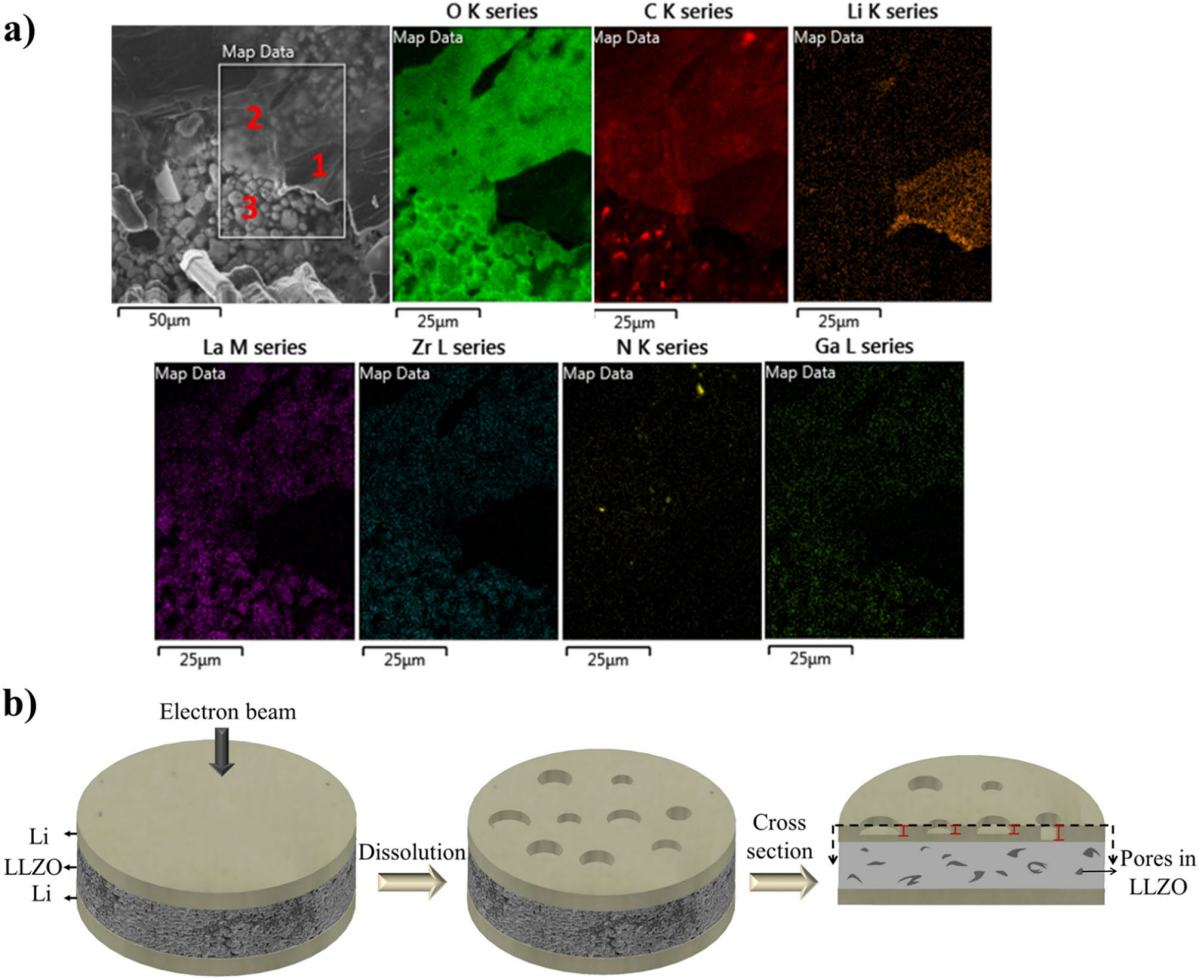
Mapping and schematic of Li thinning. (**a**) Mapping of Li surface after cycling from a region where Li thinning was observed. It is divided into three regions: Region 1 shows a high Li amount where lesser thinning occurs than that in Region 2 that shows higher amounts of C, O, La, and Zr. Region 3 is the thinnest area with the highest La and Zr contents. (**b**) Schematic of cell behavior after the dissolution. The cell cross-section shows inhomogeneous Li dissolution on different electrode regions.

Figure 3a shows the mapping of one region of metallic lithium after cycling. This mapping area is divided into three regions with different thicknesses. Region 1 has the highest amount of Li, indicating the least change in thickness. In region 2, high amounts of C and O, as well as some amounts of La and Zr from the LLZO electrolyte, were detected, indicating the thinning of Li in this part. Region 3 has the highest La and Zr contents; the SEM image shows the highest dissolution of Li in this region where even the LLZO morphology can be observed. The inhomogeneous dissolution on the entire surface of Li could be related to the crystallographic orientations of different Li grains. Some crystallographic orientations could be more susceptible to Li dissolution than others^25^. The end of the cycling curve (Figure S3) also shows significant voltage fluctuations, indicating the consumption of Li electrode and the consequent loss of contact. A schematic of the inhomogeneous dissolution of Li resulting in the thinning of various regions of the Li electrode with different thicknesses is presented in Fig. 3b (in this figure all electrodes are drawn with the same size to show the behavior of the cell during cycling better and easier to follow).

### Bump and mossy morphology dendrite

Observations show the inhomogeneous Li deposition in the form of mossy dendrites, needle dendrites, and bump morphology (Fig. 1e–h). Figure S5 shows the SEM images of some areas on the Li surface with bump and mossy morphologies. These morphologies were mainly observed close to the regions where lithium metal becomes thinner. Inhomogeneous Li deposition might be responsible for lowering the efficiency of the cell, damaging the Li surface, and resulting in a possible short circuit. Figure S5a,f show two mossy dendrites that were able to tear open the Li from the thinned region (dendrite 1) and pierce through the thinned Li (dendrite 4) where less pressure was applied. Monroe and Newman^3^ reported that if the separator’s shear modulus is more than twice that of Li, a more uniform electrodeposition can be achieved and less roughening is observed. Lithium and LLZO have shear moduli of 3.4 GPa^26^ and approximately 55 GPa^11,12^, respectively, that satisfy Monroe and Newman’s criterion. Local Li deposition and dendrite growth in these cells, however, are still observed. To overcome inhomogeneous deposition, the sole use of Monroe and Newman’s criterion is insufficient^26,27^. When the electrolyte is porous, it is still possible to observe dendrites^27^. In order to achieve a uniform lithium metal deposition both the mechanical properties of the electrolyte and the ion transport must be accomplished^26^. Tikekar et al.^26^ investigated the effect of both electrochemical and mechanical properties of the electrolyte on Li electrodeposition. They suggested three methods to achieve a more uniform electrodeposition: (a) immobilization of anions, (b) use of electrolytes with a high shear modulus, and (c) cycling at low current densities.

The main origin of inhomogeneous deposition is Li surface roughening during cycling, which results in the non-uniform current density distribution on the surface. In the case of polymer electrolytes, the polymer deformation causes variation in the concentration of lithium ions and thus ionic conductivity^26,28^. This phenomenon alters the current density on the electrode, where regions with higher conductivities encounter higher current densities that results in local deposition in these regions^26^. Tikekar et al.^26^ reported that the immobilization of anions in the electrolyte could reduce the electric field close to Li that facilitates dendritic deposition. When using polymer electrolytes, Monroe and Newman’s^3^ model introduces a new stability parameter that describes deposition and roughness. When the stability parameter is positive, a faster deposition is observed on the peaks of the deformed surface, resulting in unstable deposition and further surface roughness^3^. They also indicated that when the shear modulus of the electrolyte is greater than twice that of Li, the electrodeposition is stabilized because the parameter is negative^3^. Although ceramic electrolytes satisfy the shear modulus criterion and no electrolyte deformation occurs, unstable and non-uniform deposition is still observed. This could be the result of the inhomogeneous dissolution of Li during cycling, resulting in a rough Li electrode surface. The thinned regions are similar to the valleys in Li metal polymer batteries, and the adjacent thick regions are similar to the peaks. The exchange current density is higher near the peaks, causing a faster Li deposition. Tsai et al.^12^ also reported that inhomogeneous dissolution and deposition could result in non-uniform contacts between the ceramic and Li, causing high interface resistance and high current densities, which makes these regions susceptible to dendrite growth. Marbella et al.^7^ also reported the inhomogeneous deposition and dissolution of Li to be due to inhomogeneous contact between the electrodes. In this study, although the observation of long dendrites could be correlated to the inhomogeneous pressure on the Li electrode facing the beam, inhomogeneous Li deposition and bump and mossy morphologies cannot be fully eliminated even in the presence of homogeneous pressure. This is because the foregoing are the result of inhomogeneous Li dissolution and uneven distribution of current density on the Li electrode. In fact, the bump morphologies are original Li surface regions with new Li depositions beneath them that are unable to break the surface dendrites.

### Needle morphology dendrite

**Figure 4:**
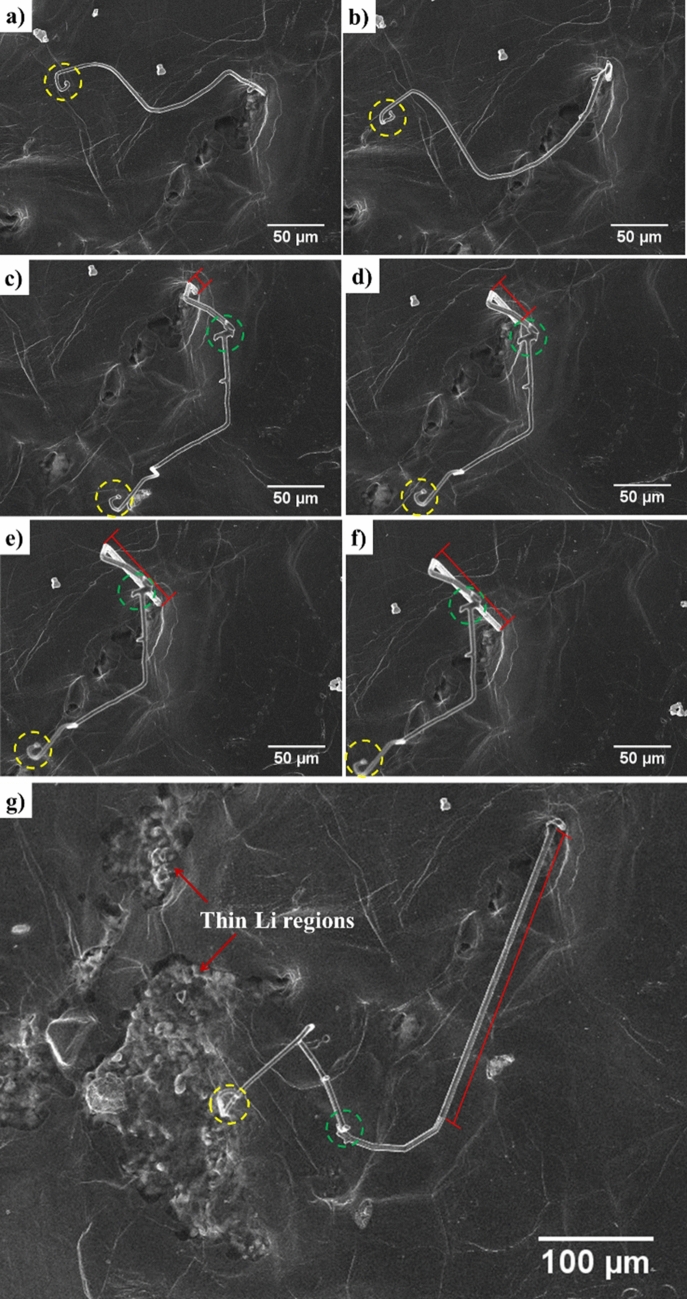
SEM images of the cell corresponding to Video S2 at + 20 µA after (**a**) 93 h, (**b**) 95 h, (**c**) 97 h, (**d**) 100 h, (**e**) 104 h, (**f**) 107 h, and (**g**) 119 h of cycling. The red lines show the change in dendrite length from the base. The yellow and green dotted circles show the regions where no change was observed. This figure shows growth from the dendrite base.

**Figure 5:**
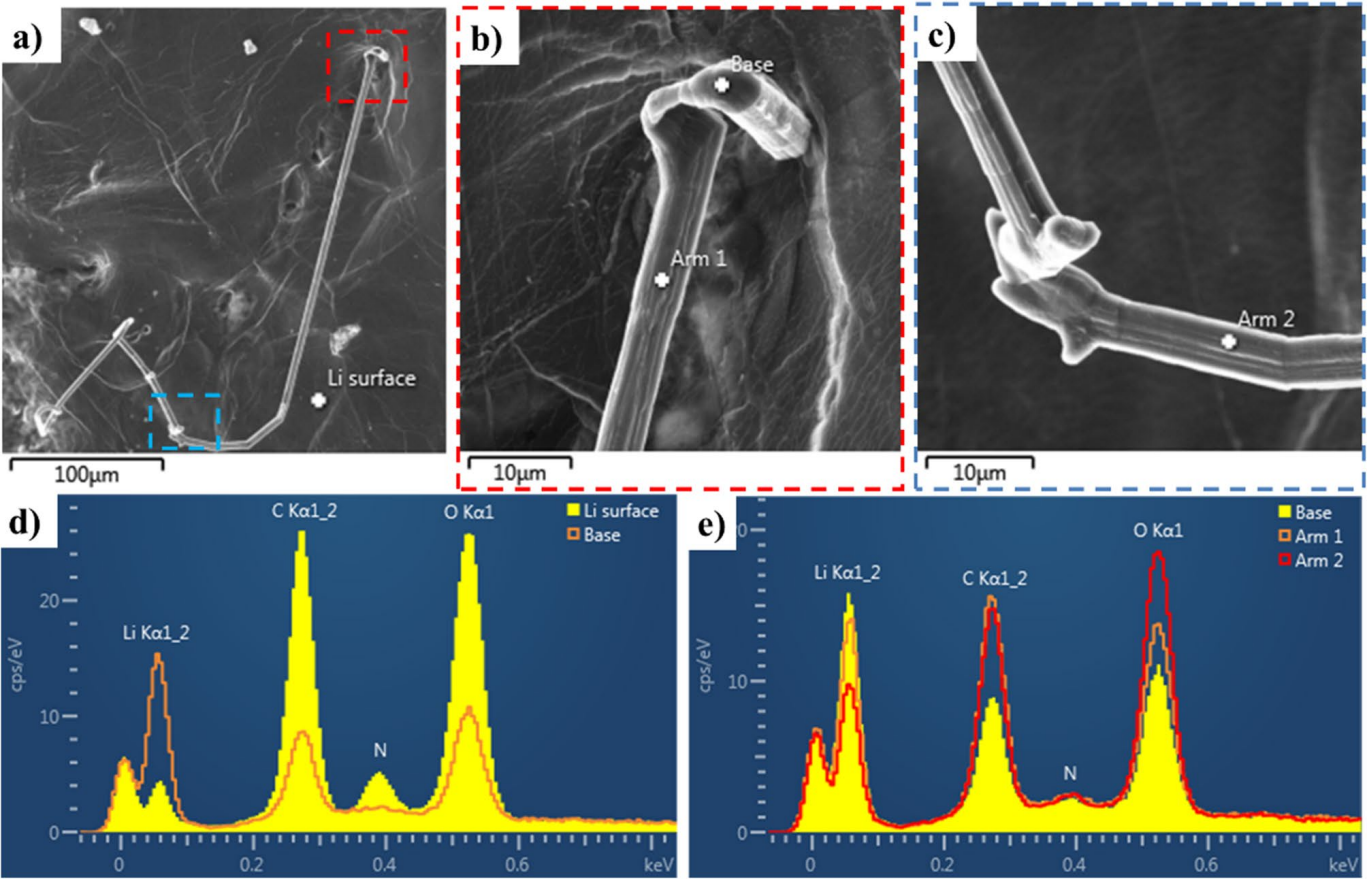
EDS analysis of the needle dendrite in Fig. 3. (**a**) SEM image of the needle dendrite; (**b**) high magnification of red dotted square in **a**; (**c**) high magnification of blue dotted square in **a**; (**d**) EDS analysis of Li surface and base with a high amount of Li at dendrite base; (**e**) EDS spectra from different parts of the dendrite showing presence of C and O.

Figure 4 shows the SEM images of the growth of a needle morphology dendrite on the Li surface when + 15 µA cm^−2^ current density was applied (Video S2). These images show that the tip (yellow dotted circle) and kink (green dotted circle) on the dendrite do not change during growth. The red line in Fig. 4 indicates the elongation of dendrite from the base. Yamaki et al.^29^ also reported dendrite growth from the base in an organic electrolyte that was compared to tin whisker growth. The growth of dendrites towards the direction where less pressure was applied (from the Li surface outward in the SEM vacuum) could be due to the compressive stress applied at the base of the dendrite as Li deposition continues in these regions. To further investigate the dendrite, a chemical analysis was conducted using EDS. Figure 5 presents the EDS analysis of the Li surface and the base and different points on the dendrite arm. The EDS analysis shows that the dendrite contains higher amount of Li than the electrode surface, which indicates the presence of fresh Li metal in the dendrite.

As indicated by the C and O contents, the dendrites are not composed of pure Li^17^. The presence of C and O could be the result of both chemical reactions during cycling and contamination. Contamination is inevitable in the SEM chamber due to polymerization of hydrocarbon molecules on the surface of the sample by the electron beam and residuals of water molecules^18,30,31^. Bessette et al.^18^ investigated the rate of O and C pick up on the Li surface at different temperatures in an SEM. In their work, a high amount of O pick up was observed in less than 20 min, whereas almost no C pick up was noted. Based on their study, it can be concluded that the C content of dendrites that are observed on the battery is mainly the result of battery cycling. A second source of C and O in the battery is represented by Li_2_CO_3_, which is present around the LLZO particles. Based on the comparison of EDS spectra of dendrites with those of Li_2_CO_3_ powder^17^, the C–O ratio in the dendrites is higher than in the Li_2_CO_3_ powder. The excess C may be caused by the presence of Li carbide (Li_x_C_y_) in the dendrites. Lithium carbide and oxide may be the result of Li carbonate reduction during cycling. Overall, it could be concluded that the dendrites are composed of a combination of Li_2_CO_3_ and Li_x_C_y_, and Li_2_O.

**Figure 6:**
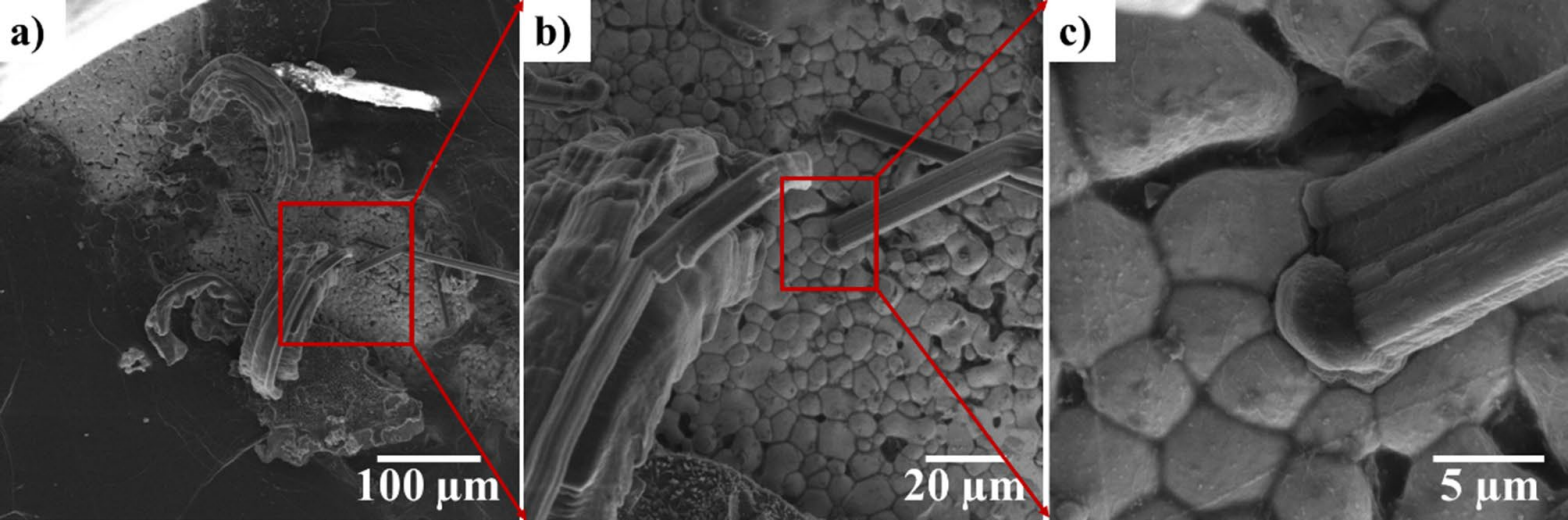
SEM images of Li surface after cycling showing dendrite growth initiation from LLZO surface on regions where Li was consumed.

Figure 6 shows the SEM images of a needle morphology dendrite that is formed on the region of the electrode where Li is consumed. The high magnification SEM image of the base shows that the dendrite initiates from the LLZO surface and grows outward rather than perforating through the electrolyte. Figure S6c–e also show two dendrites (dendrites 2 and 3) that form on a thinned Li region and pierce through Li as they grow. These images show that although LLZO provides good mechanical pressure towards dendrite perforation through the electrolytes, inhomogeneous Li deposition in the form of needle and mossy dendrites that can lower the battery efficiency are still observed. Lithium propagation through the ceramic has been observed in some studies^4–6^. Aguesse et al.^5^ detected regions containing Li metal in the cross-section of LLZO. Cheng et al.^4^ observed an opening in the cross-section of Li_6.25_Al_0.25_La_3_Zr_2_O_12_ after cycling. They attributed this to Li propagation through the grain boundaries^4^. Porz et al.^6^ reported Li propagation to be through the defects of Li_6_La_3_ZrTaO_12_.

**Figure 7:**
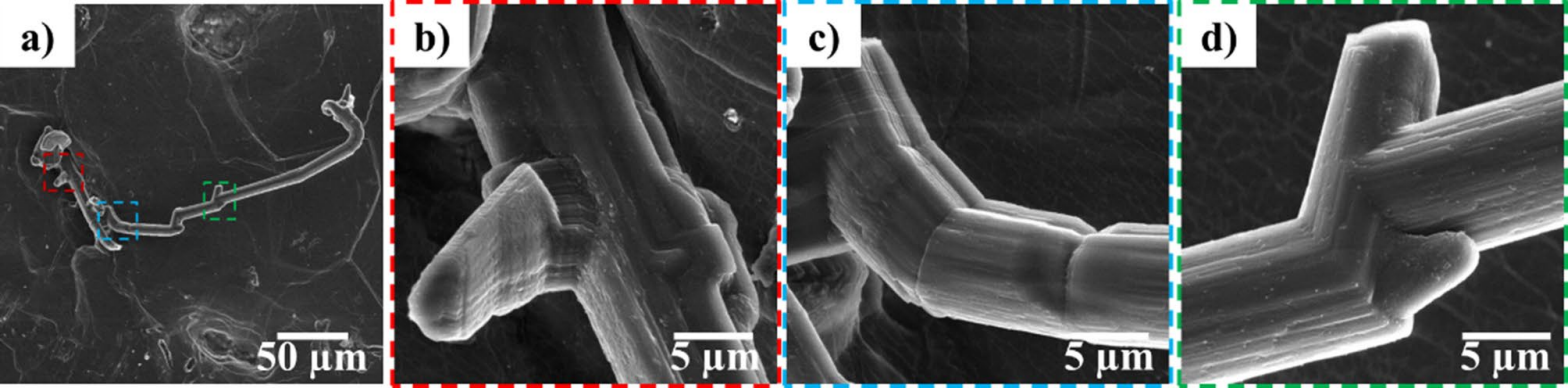
SEM images of one dendrite on Li surface and high magnification of its different regions showing growth of new arms and formation of kinks.

Dendrite formation mainly consists of two regimes—initiation and propagation^32^. Its formation, however, cannot be stopped if propagation begins^32^. In this study, inhomogeneous pressure was applied to the Li surface facing the electron beam. The variation of pressure on this Li face and the presence of ceramic on the other face could explain the dendrite growth. The foregoing only considers the mechanical properties of the electrolyte. Considering the electrochemical properties of the electrolyte, dendrite initiation is expected to occur because of inhomogeneous Li dissolution and deposition, as discussed in “Bump and mossy morphology dendrite” section. The suppression of dendrite propagation could prevent possible battery short circuits, but phenomena, such as local dissolution, deposition, and dendrite initiation, reduce battery efficiency. The use of LLZO ceramic electrolyte with applied homogenous pressure could suppress dendrite growth. To fully eliminate dendrite formation, however, the electrochemical properties of the battery should be further enhanced. High-magnification SEM images of a dendrite formed during cycling are shown in Fig. 7. These images show that the dendrites are not formed as one continuous arm but rather contain different sections and arms. This type of dendrite growth is more stable than one continuous arm because of its lower surface free energy.

### LLZO chemical analysis

**Figure 8:**
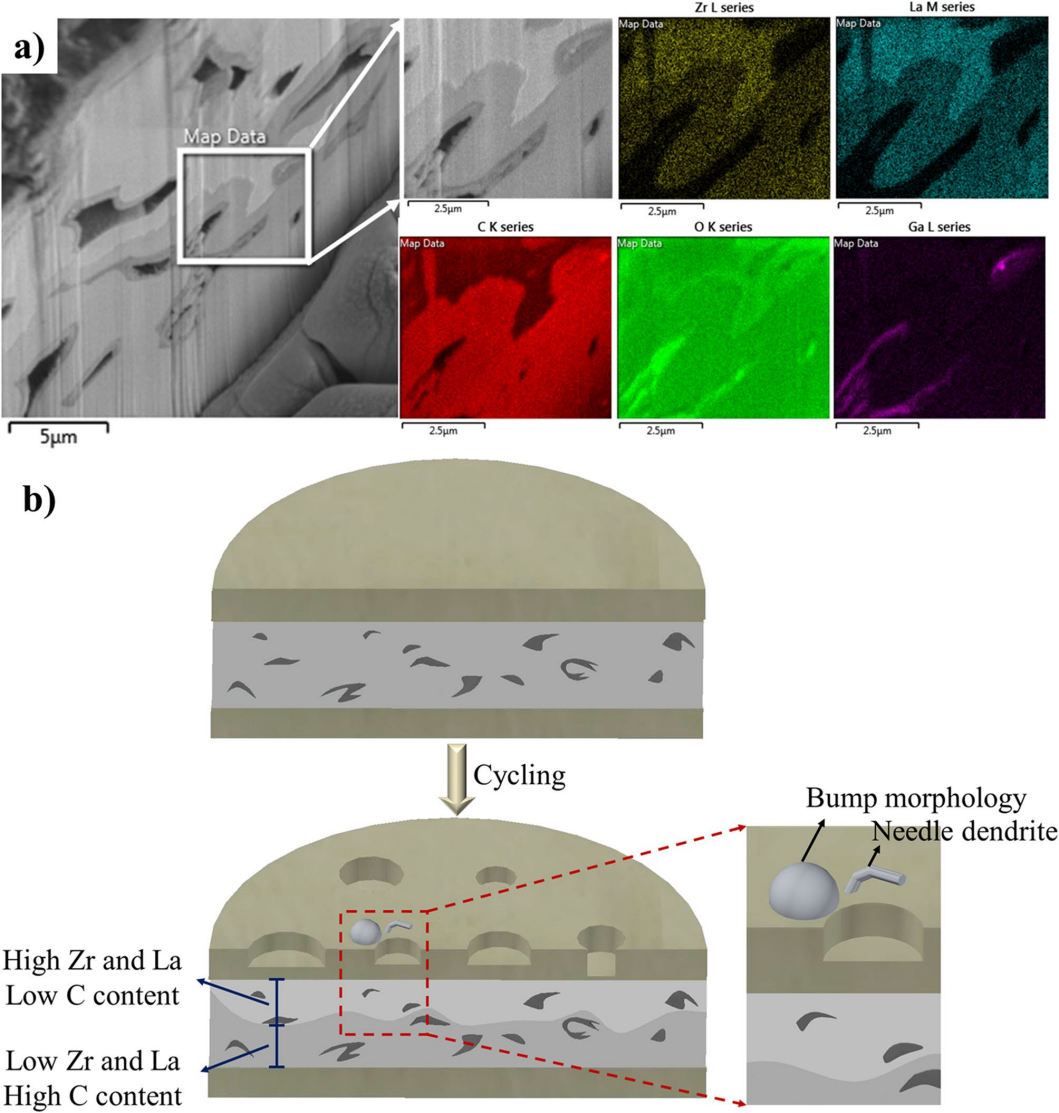
(**a**) Cross-section mapping of LLZO under the Li electrode after cycling, showing inhomogeneous distribution of Zr, La, and C, (**b**) Schematic of a cross-section of the top Li and LLZO before and after cycling. After cycling an inhomogeneous distribution of Zr, La, and C was observed, indicating La_2_Zr_2_O_7_ segregation. Close-up schematic shows one region containing bump and needle dendrite morphologies that appear close to electrode areas where Li thinning occurs.

The cross-section mapping of LLZO before cycling shows a homogeneous distribution of different elements (Figure S7). Figure 8a shows the cross-section mapping of LLZO prepared using the FIB milling after cycling. Mapping results show the inhomogeneous distributions of La, Zr, and C, where the upper part of the milled region close to the Li surface contains more La and Zr and less C. This suggests the occurrence of La_2_Zr_2_O_7_ segregation at the LLZO surface. A lower amount of C close to the LLZO surface may be the result of the consumption of C by the formation of lithium carbides. This C is from the Li_2_CO_3_ covering the LLZO particles. Higher amounts of La and Zr close to the Li electrode could be caused by the loss of Li in these regions of the ceramic and the formation of La_2_Zr_2_O_7_ resulting from the side reaction with Li_2_O. These phenomena may also be another cause of the inhomogeneous dissolution and deposition of Li. The C consumption by Li also results in the formation of mossy and needle dendrites^17^. Figure 8b shows a schematic of the cell cross-section before and after cycling, indicating the inhomogeneous Li dissolution and deposition during cycling and the chemical compositions of LLZO before and after cycling.

## Conclusion

In this work, in situ SEM is employed to analyze the LLZO/Li interface in symmetrical Li–Li cell that contains LLZO electrolyte. Dendrite growth was monitored during cycling. The dendrites cannot perforate through the LLZO electrolyte because of the mechanical force acting against their growth through this medium. However, dendrites can still form in these systems because of the presence of C as well as the inhomogeneous dissolution and deposition of Li. This results in the irreversible consumption of Li that thereby causes capacity loss during cycling. The EDS results show that dendrites are not pure Li, and they contain C and O, which could be correlated to possible chemical compositions of Li_2_CO_3_, Li_X_C_Y_, and Li_2_O. Based on the cross-section mapping of LLZO, the inhomogeneous distribution of La, Zr, and C after cycling could be caused by the Li loss close to the Li electrode and the side reaction with Li_2_O. Based on this study, the key factor resulting in dendrite growth is the LLZO/Li interface. Inhomogeneous dissolution at the interface, causes uneven distribution of current and thus inhomogeneous Li deposition. The dendrites are formed near the thin Li regions. Although LLZO has excellent mechanical properties to suppress dendrite growth, the electrochemical properties of this electrolyte should be further improved to achieve homogeneous dissolution and deposition and fully eliminate dendrite formation.

## Supplementary information

Supplementary Figures.

Supplementary Video 1.

Supplementary Video 2.
