# Supplementary material for: Direct observation of lithium metal dendrites with ceramic solid electrolyte
Source: Sci Rep. 2020 Oct 27;10:18410. doi: 10.1038/s41598-020-75456-0 (PMC7592047; doi:10.1038/s41598-020-75456-0)
Supplement: Supplementary file 1 — Supplementary Figures. [file 41598_2020_75456_MOESM1_ESM.docx]

Supporting Information

Direct observation of lithium metal dendrites with ceramic solid electrolyte

Maryam Golozar^1,2^, Hendrix Demers^1^, Sylvio Savoie^1^, Gabriel Girard^1^, Nicolas Delaporte^1^, Raynald Gauvin^2^, Abdelbast Guerfi^1^, Henning Lorrmann^3^, Karim Zaghib1,*, and Andrea Paolella^1,*^

1 Center of Excellence in Transportation Electrification and Energy Storage, Hydro-Québec, Varennes, Québec J0L 1N0, Canada

2 Department of Mining and Materials Engineering, McGill University, Montréal, Québec H3A 0C5, Canada

3 Fraunhofer-Institut für Silicatforschung ISC, Neunerplatz 2, 97082 Würzburg, Germany

*Corr. author: [zaghib.karim@hydroquebec.com](mailto:zaghib.karim@hydroquebec.com); paolella.andrea2@hydroquebec.com


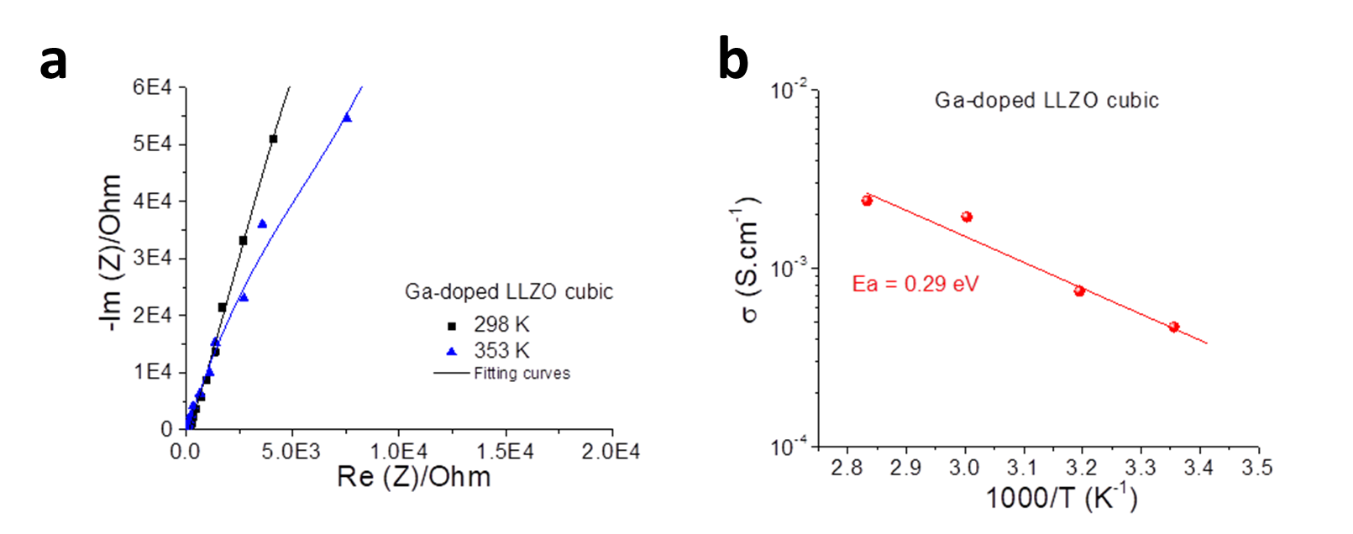


Figure S1 a) EIS plots at 25 and 80 ºC and b) Arrenhius plot of LLZO pellet.


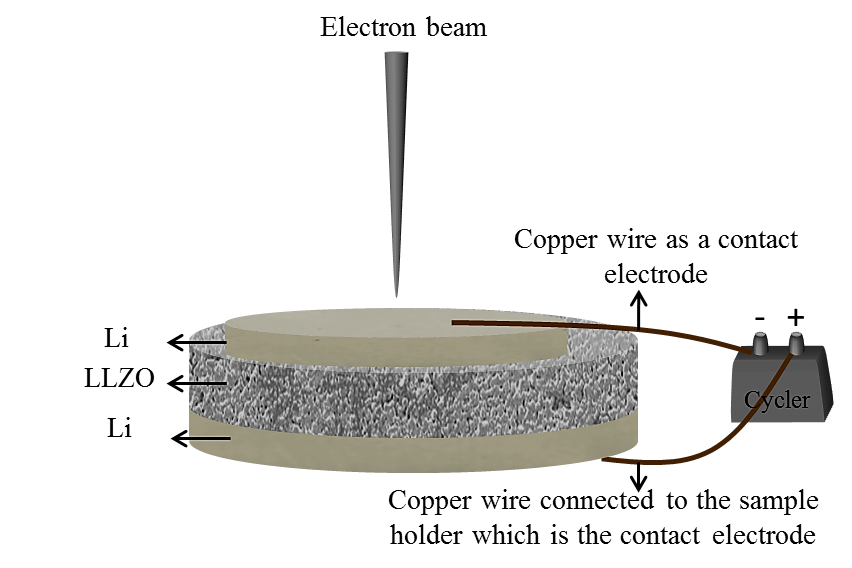


**Figure S2 Schematic of the set up. The top copper wire is a contact electrode and pushes the Li film on the LLZO with a spring. The lower copper wire is connected to the sample holder which serves as the other contact electrode. A smaller Li electrode is used on the top to have edge effect.**


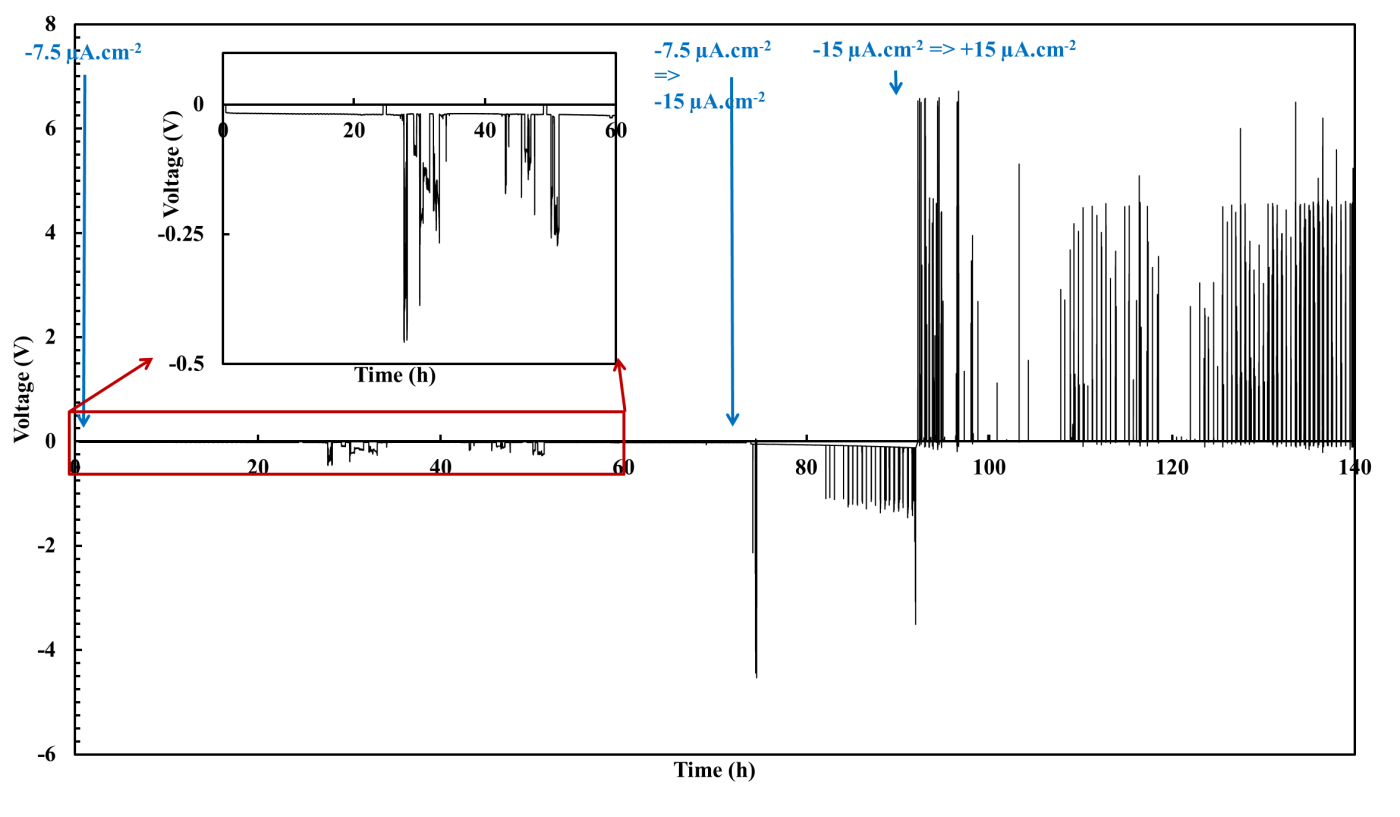


Figure S3 Cycling curve of the cell showing the time at which the current was changed.


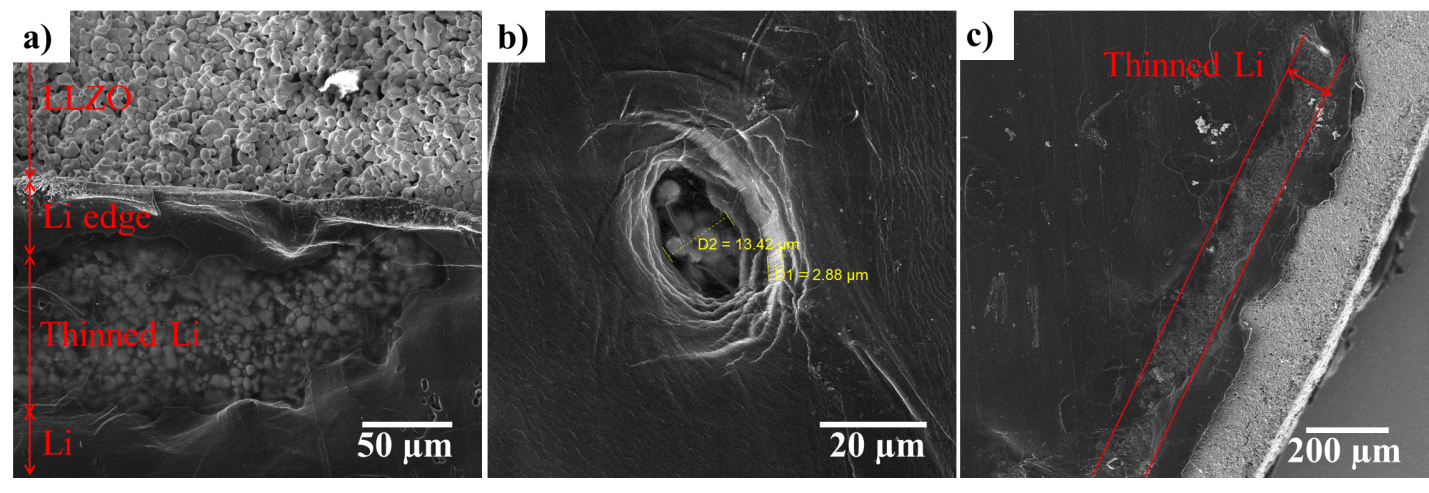


Figure S4 SEM images of the cell surface during cycling. a) SEM image of the anode edge after 3 days of cycling showing the Li anode surface, thinned Li, Li edge, and the LLZO. The thinned region is detected by comparing the morphology of the Li surface and the LLZO, b) SEM image of the Li surface after 4 days of cycling showing the diameter and the thickness of one thinned region, and c) SEM image of the Li edge after 4 days of cycling showing a ribbon of thinned region.


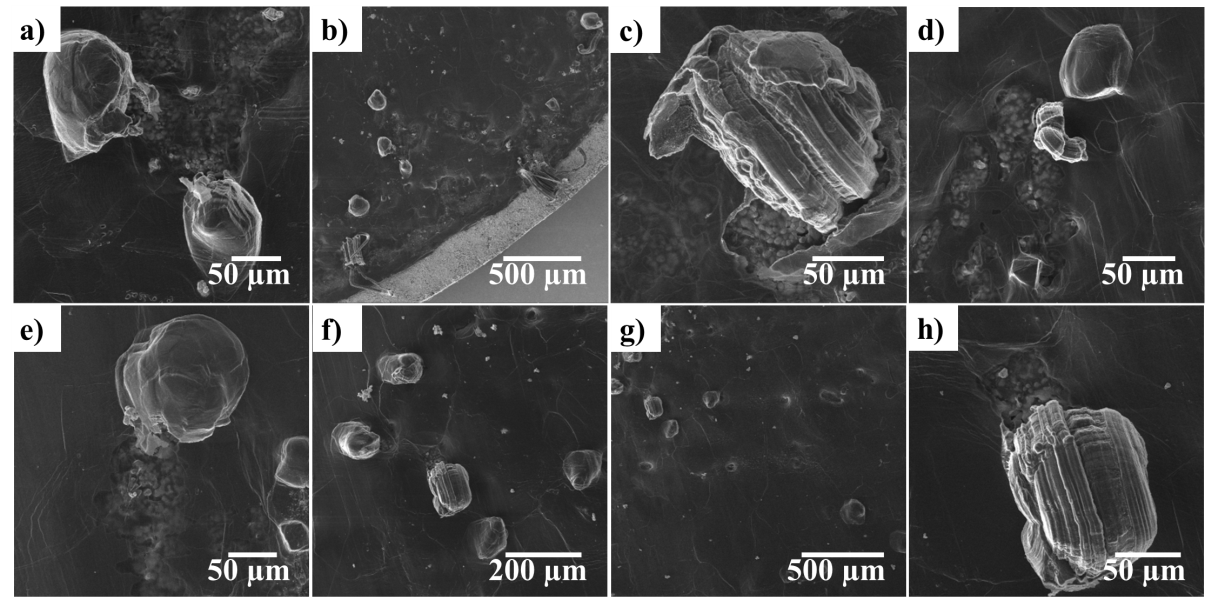


Figure S5 SEM images of different areas of the Li after cycling showing growth of mossy and needle dendrites and bumps close to the regions where Li was consumed.


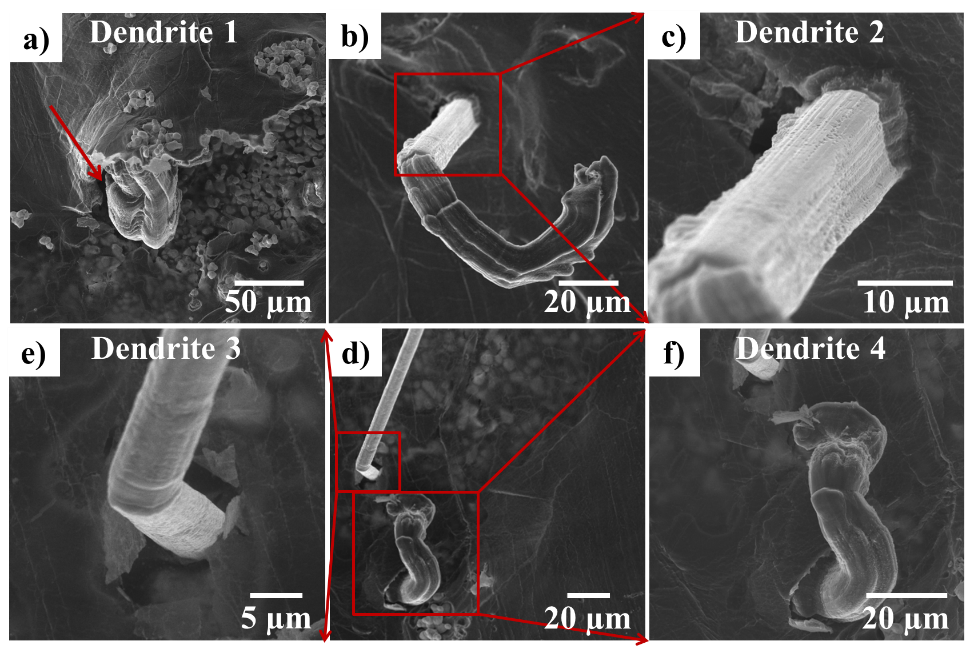


Figure S6 SEM images of dendrites with different morphologies showing ability of dendrites 2, 3, and 4 to perforate through the Li in comparison to dendrite 1 which was pushing the Li up.


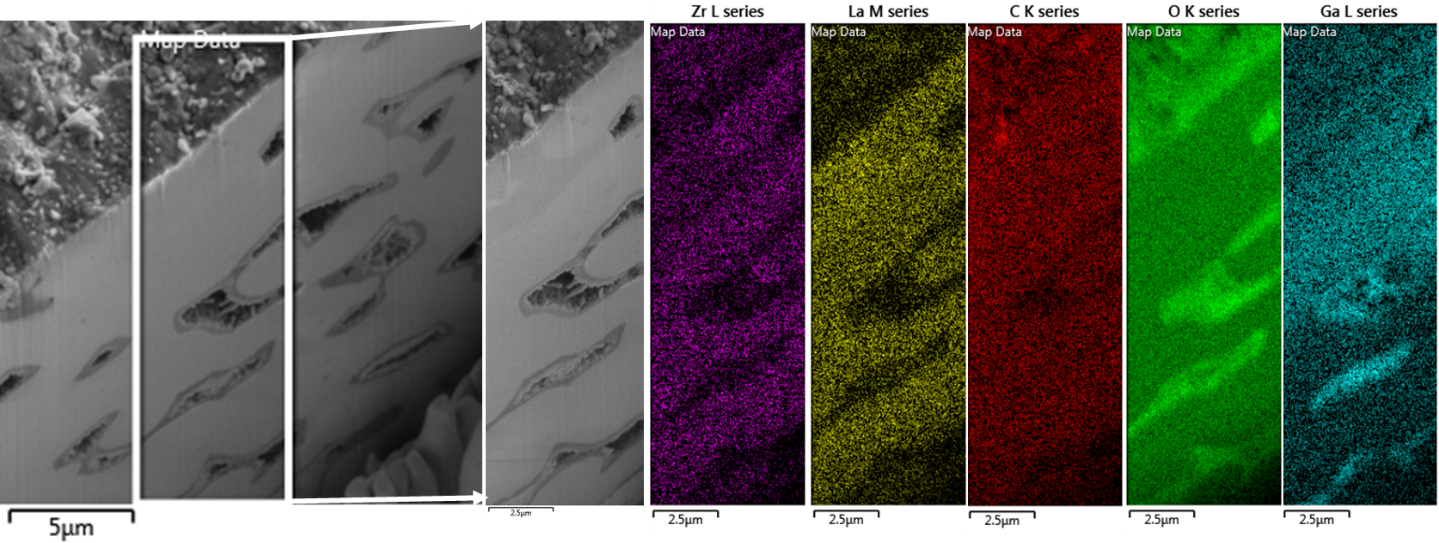


Figure S7 EDS elemental mapping of the LLZO cross section before cycling.

**Supplementary Videos:**

**Video S1:** Cycling behavior of the battery showing thinning of the Li and dendrite formation and growth.

**Video S2:** Growth of a needle morphology dendrite on the Li surface.
